# Aged Gut Microbiota Induces Mucosal Transcriptional Dysregulation, Impairing Immune Surveillance

**DOI:** 10.1111/acel.70533

**Published:** 2026-05-04

**Authors:** Fang Wu, Ming Zhang, Jianmin Wu, Zihan Wang, Yumeng Ma, Lin Dong, Le Cheng, Tengteng Ji, Chenyan Zheng, Fazheng Ren, Bing Fang

**Affiliations:** ^1^ Key Laboratory of Precision Nutrition and Food Quality, Department of Nutrition and Health China Agricultural University Beijing China; ^2^ School of Food and Health Beijing Technology and Business University Beijing China

**Keywords:** aging, *Clostridium difficile* infection, follicle‐associated epithelium (FAE), gut microbiota, intestinal mucosal immunity

## Abstract

Aging is associated with systemic immune remodeling and disease susceptibility, but its impact on intestinal mucosal immunity, particularly changes in M cells, remains largely unknown. This study aimed to investigate how aging alters intestinal mucosal immune phenotypes, specifically follicle‐associated epithelial cells (FAE) and the gut microbiota, and to identify interconnected pathways that may be exploited to maintain intestinal immune function in the elderly. Using intestinal tissue from young and aged mice, this study assessed manifestations of intestinal epithelial aging, changes in immune cells in the lamina propria, and microbial composition. Aging was associated with increased expression of senescence‐associated secretory phenotype (SASP) markers (*IL‐1β*, *TNF‐α*, *p16*) and decreased levels of tight junction proteins (*Occludin*, *Tricellulin*), suggesting epithelial barrier dysfunction. Aged mice exhibited decreased Naïve Th cells, increased Effector Th and Th17 subsets, and decreased fecal IgA. Microbiome analysis revealed enrichment of inflammatory bacteria, such as *Desulfovibrio* and *Candidatus_Saccharimonas*, and elevated dysbiosis indices. RNA sequencing of FAEs revealed 578 differentially expressed genes, including downregulation of *Gp2* and *Ccl28*, indicating impaired M cell function. Association analysis between microbiome changes and mucosal immune aging revealed that enrichment of key inflammatory bacteria may contribute to impaired M cell function and dysregulated intestinal mucosal immunity. These findings reveal a multi‐layered disruption of intestinal homeostasis during aging—comprising barrier function, immune imbalance, FAEs dysfunction, and shifts in specific microbial taxa ‐leading to increased susceptibility to pathogens. Targeting these age‐related pathways may provide strategies for maintaining intestinal immunity in the elderly.

## Graphical abstract

Aging impaired intestinal mucosal immune homeostasis by altering the epithelial barrier, lamina propria immune cells, follicle‐associated epithelium, and gut microbiota. These changes, particularly the accumulation of pro‐inflammatory bacteria in the gut microbiota, are closely associated with dysregulation of follicle‐associated epithelium gene expression and increased susceptibility to pathogens.

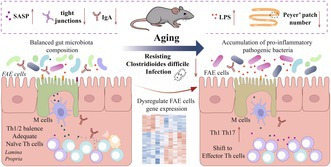

## 1 Introduction

The gastrointestinal tract serves as a critical interface between the host and the external environment (Yang et al. 2025). The intestinal mucosal barrier plays a pivotal role in immune surveillance and maintaining microbial homeostasis (Turner 2009). With aging, the integrity of this barrier declines: tight junctions become compromised (Neurath et al. 2025), and the secretion of mucins and antimicrobial peptides diminishes (Okumura and Takeda 2017). Peyer's patch (PP) and mesenteric lymph node undergo involution, with a concomitant reduction in germinal centers, leading to impaired antigen presentation (Reboldi and Cyster 2016). Additionally, immune cell dysregulation occurs in the intestinal lamina propria—macrophage phagocytic capacity declines, T cell activation becomes attenuated and aberrantly polarized, regulatory T cells (Tregs) decrease, the Th17/Treg balance is disrupted (Yan et al. 2026), and B cell‐derived immunoglobulin A (IgA) production is reduced (Zheng et al. 2022). Collectively, these changes contribute to increased susceptibility to pathogens such as *Clostridium difficile* and *norovirus* in the elderly (Bouwknegt et al. 2015), and promote chronic inflammation associated with immunosenescence (Franceschi et al. 2018). In mucosal immunity, Peyer's patches represent specialized lymphoid structures in the small intestine that mediate antigen surveillance and immune activation (Donaldson et al. 2021). These structures are overlaid by the follicle‐associated epithelium (FAE), the principal site for M cell‐mediated antigen uptake and immune initiation (Nakamura et al. 2018). However, the aging‐associated alterations in FAE within Peyer's patches, and their potential role in regulating M cell function, remain poorly characterized.

Disruptions in microbiota‐immune crosstalk are increasingly recognized as contributing factors in the pathogenesis of various immune‐mediated diseases (O'Toole and Jeffery 2015). The gut microbiota is a dynamic ecosystem co‐evolved with the host; in healthy young individuals, commensal microbes support immune homeostasis through mechanisms such as short‐chain fatty acid (SCFA) production (Xiao et al. 2025), Toll‐like receptor (TLR) signaling, and induction of Tregs (Thevaranjan et al. 2017). Aging, however, is associated with pronounced dysbiosis, marked by reduced microbial diversity, depletion of beneficial taxa (*Bifidobacterium*, *Faecalibacterium*) (Ghosh et al. 2022), enrichment of pathobionts (*Enterobacteriaceae*), altered microbial metabolites, and diminished SCFA levels‐all of which contribute to immune aging (Ahmad Fadzuli et al. 2024). Recent studies have shown that specific microbes, such as *Segmented Filamentous Bacteria* (Tanoue et al. 2010), can induce FAE remodeling and M cell differentiation (Lai et al. 2020). Whether the aged microbiota retains this capacity or instead promotes pathogenic FAE reprogramming remains an open and critical question.

Here, we employed a multimodal approach to investigate how aging reshapes the interaction between gut microbiota and transcriptional networks within the FAE. By integrating RNA sequencing data of the gut microbiota and FAEs from young and aged mice, we identified age‐associated microbial taxa that were closely associated with genes for M cell differentiation and function. Our results reveal a link between the proliferation of pathogenic bacteria in the gut and dysregulation of the FAE gene network during aging. By elucidating the triadic interactions among the microbiota, epithelium, and immune system, this study enhances our understanding of intestinal barrier deterioration with age and identifies potential targets for restoring mucosal immune function in the elderly.

## 2 Materials and Methods

### 2.1 Animals and Experimental Design

C57BL/6J mice (age 3 months and 24 months) were purchased from SPF Biotechnology Co. Ltd. (Beijing, China) and bred in a pathogen‐free environment in the Laboratory Animal Center of China Agricultural University (Beijing, China). All mice were kept in standard cages and same conditions (on a 12/12 h light/dark cycle) with free access to drinking water and food. All experiments involving animals were conducted according to the ethical policies and procedures, followed National Research Council's Guide for the Care and Use of Laboratory Animals and approved by the Ethics Committee of China Agricultural University (AW40113202‐5‐2). Intestinal tissues and fecal samples were collected at the time of sacrifice and stored at −80°C for subsequent microbiome and biochemical analyses.

In the infection trial, 3‐month‐old and 24‐month‐old male mice received either vehicle or 200 μL of *Clostridioides difficile* VPI 10463 ( *C. difficile* , 1.0 × 10^9^ CFU/mL) by oral gavage following 3 days of antibiotic cocktail treatment in drinking water and 1 day of recovery (Wu et al. 2024). *C. difficile* was cultured in brain heart infusion medium, and its concentration was determined by serial dilution and plate counting. After 7 days, the mice were sacrificed to assess differences in susceptibility to *C. difficile* infection.

### 2.2 Detection of Fecal IgA and Lipopolysaccharides (LPS) Levels

Fecal IgA and LPS levels were measured using Mouse IgA ELISA Kits (EK274, MULTI SCIENCES) and LPS ELISA kits (E‐EL‐0180, Elabscience). Briefly, approximately 100 mg of fecal samples were homogenized in 1 mL phosphate buffered saline (PBS) and centrifuged at 12,000 rpm for 10 min at 4°C. The supernatants were diluted 1:10 to ensure measurements fell within the standard curve range. Samples and IgA or LPS standards were added to antibody‐coated wells and incubated for 1.5 h. After washing the plates five times with wash buffer, HRP‐conjugated enzyme solution was added and incubated at 37°C for 1 h. Plates were again washed, followed by the addition of TMB substrate and incubation at room temperature for 15 min in the dark. The reaction was stopped with stop solution, and absorbance was measured at 450 nm. Each sample was analyzed in triplicate. Concentrations were calculated based on standard curves generated from the standards' absorbance values.

### 2.3 Isolation of the Small Intestine Lamina Propria

The intestinal segments were thoroughly rinsed in pre‐cooled PBS to remove connective tissue, then cut longitudinally. After scraping off the villi, the tissue was chopped into 0.3 cm pieces and incubated in predigestion solution (PBS with 5 mM EDTA) at 37°C, 220 rpm for 25 min to detach epithelial cells. Following several washes and filtration (100 μm), the remaining tissue was digested in DMEM/F12 medium (Thermo Fisher Scientific) containing 300 U/mL collagenase II (Thermo Fisher Scientific) at 37°C, 110 rpm for 30 min. Finally, after passing through a 40 μm sieve, lamina propria cells were collected by centrifugation at 500*g* for 5 min at room temperature (Liu et al. 2024).

### 2.4 Flow Cytometry Analyses

Intestinal lamina propria cells were washed with PBS and resuspended in RPMI‐1640 medium (Thermo Fisher Scientific) at 1 × 10^6^ cells/mL. For intracellular cytokine staining, prior stimulation and secretion blockade were performed by adding 2 μL of Cell Activation Cocktail (containing Brefeldin A, BioLegend) per 1 mL of cell suspension, followed by incubation for 5 h at 37°C in 5% CO_2_. Cells were then washed with Cell Staining Buffer (BioLegend) and stained with fluorescent antibodies against CD45, CD3, and CD4 for 30 min at 4°C. After fixation and permeabilization with Cyto‐Fast Fix/Perm Buffer (BioLegend), intracellular IL‐4, IL‐17A, and IFN‐γ were stained. For only surface receptor staining, no stimulation was required. Cells were directly stained with fluorescent antibodies against CD45, CD3, CD4, CD44, and CD62L for 30 min at 4°C after washing. Following two final washes, samples were acquired on a BD FACS Aria III (BD Biosciences), and data were analyzed using BD FACS Diva and FlowJo 10.8 software. Gating strategies included forward and side scatter, singlets, and live CD45^+^ cells. We label intestinal lamina propria immune cells with CD45 and T cells with CD3, then simultaneously define CD4 and IL‐17A double positivity to distinguish Th17 in one figure (similarly, IL‐17A is replaced by IFN‐γ, and IL‐4 is used to distinguish Th1 and Th2 respectively). Specific populations were identified as follows: CD4^+^ T cells (CD3^+^CD4^+^); Naïve (CD44^−^CD62L^+^), Effector (CD44^+^CD62L^−^), and Memory (CD44^+^CD62L^+^) subsets; Th1 (IFN‐γ^+^), Th2 (IL‐4^+^), and Th17 (IL‐17A^+^) (Cignarella et al. 2018). Antibody information used for flow cytometry are listed in Table S1.

### 2.5 RNA Extraction and Real‐Time Quantitative Polymerase Chain Reaction (RT‐PCR) Analysis

Total RNA was extracted from the intestinal tissue using TRIzol (Invitrogen, USA) and isolated by TRIzol RNA extraction. Total RNA (500 ng/μL) was used for reverse transcription with All‐In‐One 5× RT MasterMix (G592, abm). RT‐PCR was performed using PowerUP SYBR Green Master Mix (A25742, Thermo Fisher Scientific) with cDNAand primers. The primer sequences listed in Table S2. The primers were synthesized by Sangon (Beijing, China). The relative expression levels were calculated compared with the control group, with GAPDH, using the 2^−ΔΔCT^ method.

### 2.6 Gut Microbiota 16S Ribosomal RNA (rRNA) Sequencing

Intestinal content samples were collected from mice and stored at −80°C. Samples were sent to Majorbio Bio‐Pharm Technology (Shanghai, China) for DNA extraction and 16S rDNA sequencing. Total microbial genomic DNA was extracted by the PF Mag‐Bind stool DNA Kit (Omega Bio‐tek, Georgia, USA). The hypervariable region V3‐V4 of the bacterial 16S rRNA gene were amplified with primer pairs 338F (5′‐ACTCCTACGGGAGGCAGCAG‐3′) and 806R (5′‐GGACTACHVGGGTWTCT AAT‐3′) by ABI GeneAmp 9700 PCR thermocycler (ABI, CA, USA). Purified amplicons were pooled in equimolar amounts and sequenced on an Illumina PE300/PE250 platform (Illumina, USA). Raw reads were quality‐filtered with fastp v0.19.6 (Chen et al. 2018), merged using FLASH v1.2.11 (Magoc and Salzberg 2011). Bioinformatic analysis was conducted on the Majorbio Cloud platform (https://cloud.majorbio.com).

Beta diversity was visualized using Principal Coordinate Analysis (PCoA) based on Bray–Curtis dissimilarity matrices. To statistically evaluate the differences in overall microbial community structure between groups, Permutational Multivariate Analysis of Variance (PERMANOVA, Adonis) was performed using the adonis2 function from the R‐3.3.1 (vegan), with 999 permutations. Circos plots visualized sample‐species relationships.

Linear discriminant analysis Effect Size (LEfSe) was performed to identify significantly enriched taxa (LDA score > 2, *p* < 0.05), which inherently utilizes the Kruskal‐Wallis test among classes and the Wilcoxon test between subclasses followed by LDA. Furthermore, to complement the LEfSe results and independently validate the differential abundance of specific key genera between the young and aged groups, standalone non‐parametric Wilcoxon rank‐sum tests (Mann–Whitney U tests) were conducted using the stats package in R (version 3.3.1). The *p*‐value < 0.05 was considered statistically significant.

To quantitatively evaluate the overall health status and the degree of microbial dysbiosis in the gut microbiome, the Gut Microbiome Health Index (GMHI) and Microbiome Dysbiosis Index (MDI) were calculated. The GMHI was computed based on species‐level taxonomic profiles following the methodology described (Gupta et al. 2020). Briefly, microbial species were classified as “health‐prevalent” or “health‐scarce” based on their prevalence in the baseline (young) versus the altered (aged) groups. A species was considered “present” if its relative abundance was ≥ 1.0 × 10^−5^. The selection of these species relied on predefined thresholds for both prevalence fold‐change (ranging from 1.2 to 2.0) and prevalence difference. This index provides a robust stratification of health status independent of ecological metrics like alpha diversity. The GMHI analysis was implemented using Python (version 2.7.10). The MDI was determined to quantify the severity of microbial dysbiosis, where a higher MDI value indicates a greater degree of microbiota disruption (Gunathilake et al. 2020). First, the fold change of each genus was calculated as the ratio of its mean relative abundance in the aged group to that in the young baseline group. Genera were subsequently categorized as either increased or decreased in the aged group. The MDI was then calculated as the base‐10 logarithm of the ratio of the total abundance of genera increased in the aged group to the total abundance of genera decreased in the aged group. The MDI calculation was performed using Python (version 2.7.10) and the vegan package (version 2.4.3) in R software (version 3.3.1).

Microbial phenotypes were predicted using BugBase (https://bugbase.cs.umn.edu/index.html). Briefly, the operational taxonomic units (OTUs) were first normalized by their predicted 16S rRNA gene copy numbers. Subsequently, microbial phenotypes were predicted utilizing the tool's pre‐calculated trait files. The predicted phenotypes were classified into seven major categories: Gram‐Positive, Gram‐Negative, Biofilm‐Forming, Pathogenic, Mobile Element‐Containing, Oxygen‐Utilizing (including Aerobic, Anaerobic, and Facultatively Anaerobic), and Stress‐Tolerant. It should be noted that these phenotypic categories are not mutually exclusive, as a single microbial taxon can possess multiple traits simultaneously.

### 2.7 Isolation and Preparation of FAE From Peyer's Patches (PPs)

FAE and villus epithelium (VE) sheets were isolated using an EDTA‐assisted microdissection method (Donaldson et al. 2021). Briefly, intestinal segments containing Peyer's patches were dissected and incubated in ice‐cold Hank's balanced salt solution (HBSS) containing 30 mM EDTA for 15 min to disrupt intercellular junctions. Under a stereomicroscope, the dome regions of Peyer's patches were carefully microdissected, and the FAE layer was gently separated from the underlying tissue using fine needles, followed by trimming of residual villus structures. For VE isolation, adjacent 5 mm ileal segments devoid of Peyer's patches were processed identically. The isolated FAE and VE sheets, along with intact ileal tissue sections, were immediately frozen and stored at −80°C for subsequent RNA extraction and analysis.

### 2.8 FAE Transcriptome Profiling (RNA‐Seq)

Total RNA extraction and sequencing: total RNA was isolated FAE samples using the QIAzolLysisReagent (Qiagen) with gDNA elimination, followed by library preparation with the Illumina Stranded mRNA Prep (Illumina) incorporating RiboZero Gold rRNA depletion; after quality control, libraries were pooled and sequenced on NovaSeq Reagent Kit (NovaSeq X Plus) (Illumina). Raw reads were adapter‐ and quality‐trimmed (Q30, min length 50 bp) using Cutadapt, aligned to the *Mus musculus* GRCh38 genome with STAR (default parameters, paired‐end mode), and quantified via featureCounts (reverse strand, mapping quality ≥ 10); FPKM values were generated using edgeR with gene lengths defined as non‐redundant exonic bases, normalized by effective library size. To identify differential expression genes (DEGs) between two different samples, the expression level of each transcript was calculated according to the transcripts per million reads (TPM) method. RSEM was used to quantify gene abundances (Li and Dewey 2011). Essentially, differential expression analysis was performed using the DESeq2 (Love et al. 2014). Genes with |log_2_FC| ≥ 1 and False Discovery Rate (FDR) < 0.05 were considered significantly differently expressed.

Functional enrichment analysis was performed to identify the primary biological functions and pathways associated with the DEGs. All expressed genes identified in the transcriptomic dataset were used as the background gene set. Gene Ontology (GO) enrichment analysis was conducted using the Python package GOatools (https://github.com/tanghaibao/GOatools). The statistical significance of the enrichment was determined using Fisher's exact test. To control the false positive rate, *p*‐values were adjusted using multiple testing correction methods, including the FDR. A GO term was considered significantly enriched when the FDR‐adjusted *p*‐value was < 0.05. Kyoto Encyclopedia of Genes and Genomes (KEGG) pathway enrichment analysis was performed using the scipy package in Python (https://scipy.org/). Similar to the GO analysis, Fisher's exact test was applied to calculate the significance of pathway enrichment. The *p*‐values were adjusted using the Benjamini‐Hochberg (BH) FDR method, and pathways with a corrected *p*‐value < 0.05 were defined as significantly enriched.

Weighted gene co‐expression network analysis (WGCNA) was performed using the WGCNA R package (v1.72‐1) in R (v4.x). Genes with low expression variability were filtered prior to analysis. Pairwise gene correlations were calculated using the Spearman method. A signed co‐expression network was constructed with a soft‐thresholding power (β) of 9 to approximate scale‐free topology. The adjacency matrix was transformed into a topological overlap matrix (TOM), and genes were hierarchically clustered using average linkage. Gene modules were identified using the dynamic tree cut algorithm (cutreeDynamic, method = “hybrid”) with a minimum module size of 30 genes. Modules with highly correlated eigengenes were merged using a merge cut height of 0.25. Module‐trait associations were evaluated by correlating module eigengenes with continuous phenotypic traits using Spearman correlation. Corresponding *p* values were calculated, and modules with *p* < 0.05 were considered significantly associated with the traits of interest. Hub genes within significant modules were identified based on high module membership and gene significance.

### 2.9 Multi‐Omics Integration and Correlation Analysis

Spearman correlation analysis was used to evaluate the relationship between LPS, IgA concentrations, PP number and genus‐level microbial abundance, and the relationship between the relative abundance of specific genera and key differentially expressed genes. We performed non‐parametric Spearman rank correlation analysis. The resulting correlation coefficients were visualized as heatmaps, where color gradients indicate the strength and direction of the associations. To control for false positives in multiple comparisons, the raw *p*‐values were adjusted using the FDR (Benjamini‐Hochberg) method. The correlation analyses and heatmap visualizations were conducted using the pheatmap package in R software (version 3.3.1).

Mantel tests were performed to assess the association between the overall microbial community structure and the host gene expression matrix. Significance was evaluated using permutation tests (*n* = 999 permutations). This analysis evaluated the correlation between the overall microbial community structure (based on Bray‐Curtis distance) and the expression profiles of gene sets related to intestinal aging and barrier function (based on Euclidean distance).

To validate the clinical significance of age‐related FAE features, we analyzed the human gut IBD transcriptome dataset obtained from the Inflammatory Bowel Disease Transcriptomic Database (IBDTransDB). We analyzed the expression levels of the key age‐related FAE gene *Gp2* in GSE83687. Statistical significance was assessed using unpaired Student's *t*‐test, * 0.01 < *p* ≤ 0.05, *** 0.0001 < *p* ≤ 0.001. The resulting differentially expressed genes were visualized via volcano plots using R software.

### 2.10 Statistical Analysis

Statistical analysis was performed using the GraphPad: Prism 9 software. Comparisons between two groups were made by an unpaired two‐tailed Student's *t*‐test. Multiple comparisons of one‐variable data were carried out by one‐way analysis of variance.

## 3 Results

### 3.1 Mucosal Immune Dysregulation Occurs in the Intestine of Aged Mice

**FIGURE 1:**
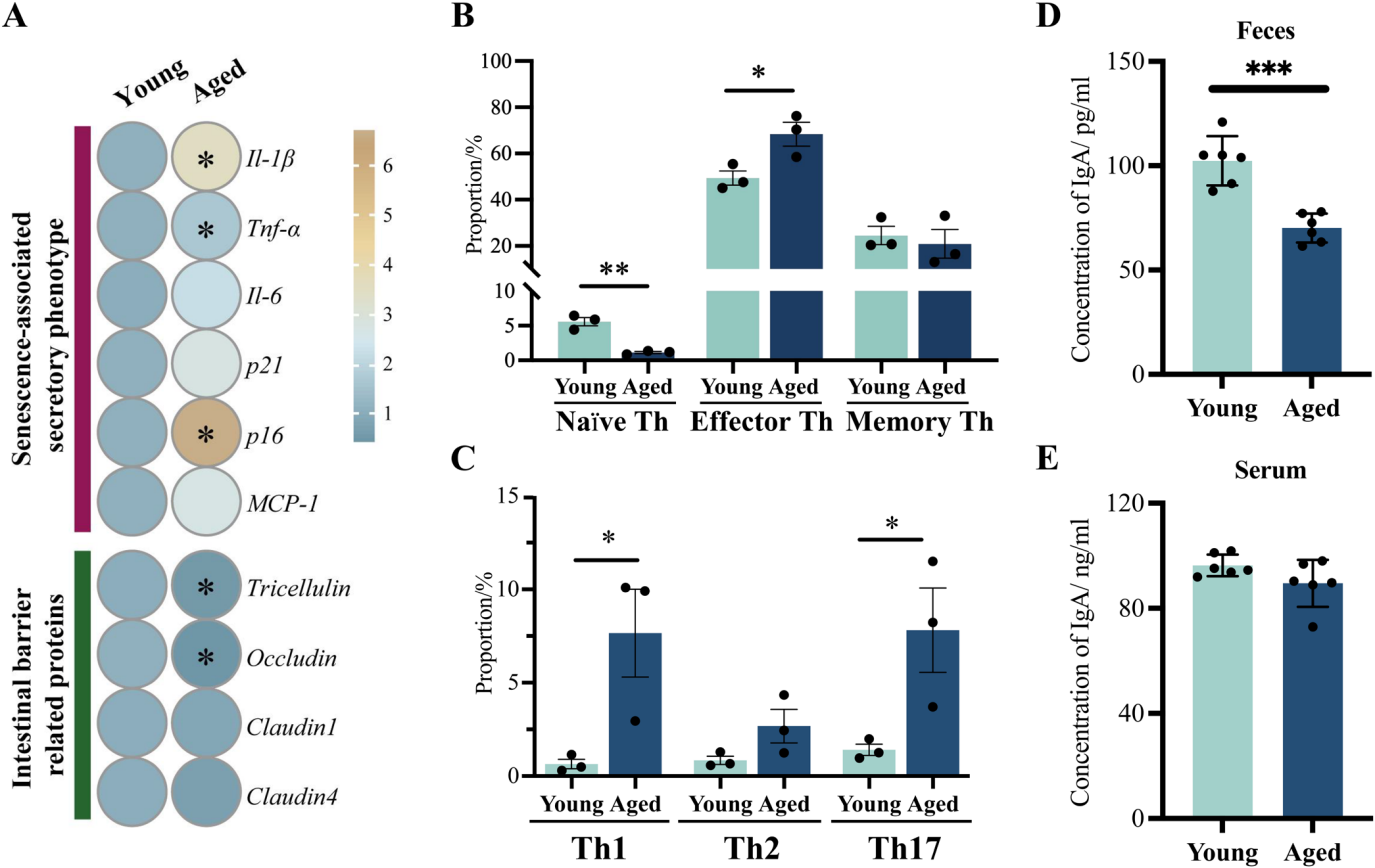
Aging‐related changes in the intestinal barrier and immune cell spectrum. (A) Relative expression levels of intestinal aging‐associated secretory phenotypes and barrier‐related markers. (B) Quantification of Naïve, Effector and Memory Th cells in the intestinal lamina propria. (C) Quantification of Th1, Th2, and Th17 cells in the intestinal lamina propria. (D, E) IgA concentrations in the feces (D) and serum (E) of young and aged mice. *p < 0.05, **p < 0.01, ***p < 0.001; two‐tailed Student's *t*‐test.

**FIGURE 2:**
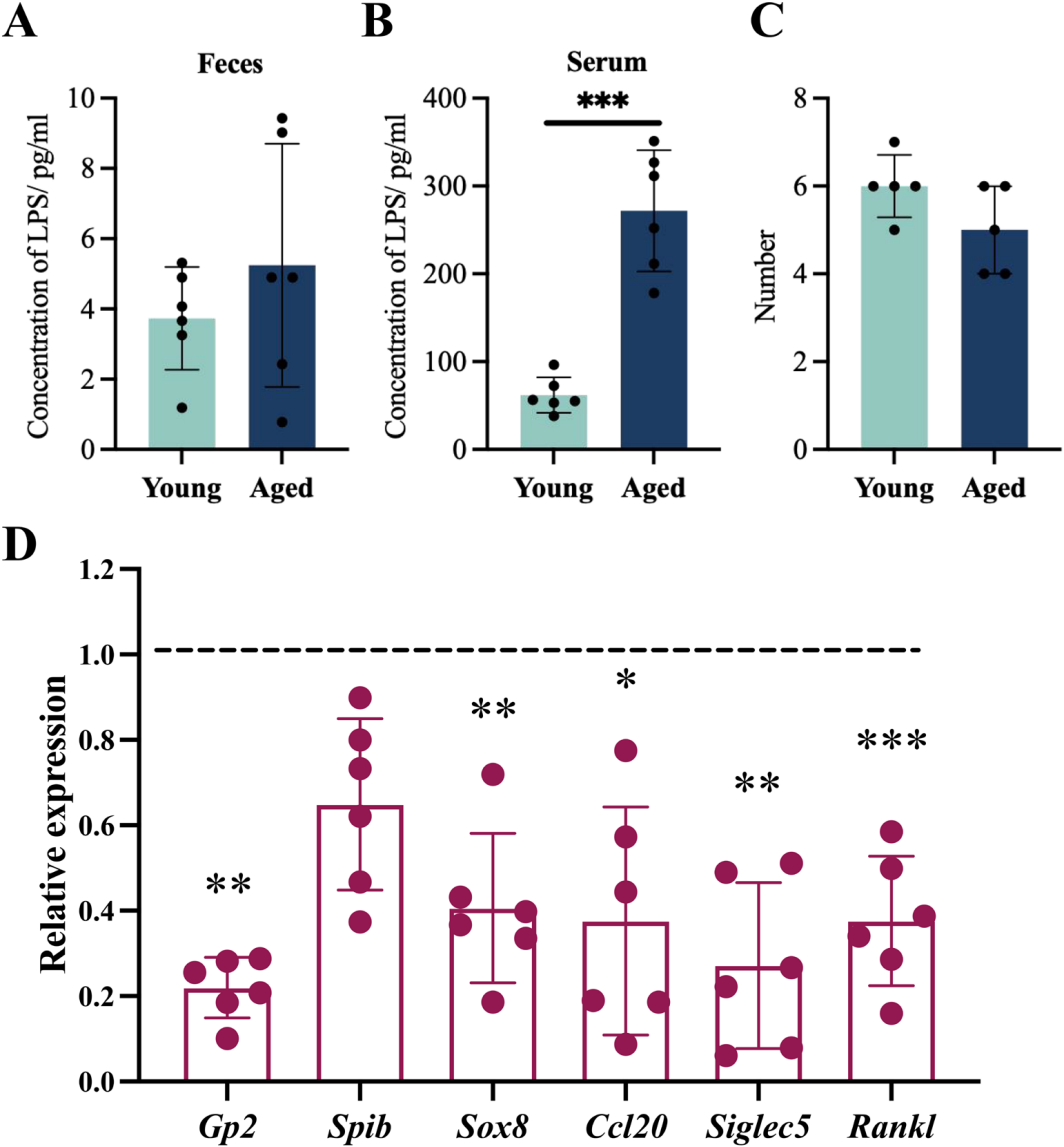
Aging‐related changes in intestinal M cell differentiation and functional markers. (A, B) LPS concentrations in the feces and serum of young and aged mice. (C) Number of Peyer's patches in the intestines of young and aged mice. (D) Relative expression levels of M cell differentiation and functional markers. *p < 0.05, **p < 0.01, ***p < 0.001; two‐tailed Student's t‐test.

To explore the effects of aging on intestinal mucosal immune function, we analyzed aging‐related changes including intestinal barrier, intestinal lamina propria, and Peyer's patch (Figures 1 and 2). We analyzed the expression changes of key senescence‐associated secretory phenotypes (SASP) (*IL‐1β*, *TNF‐α*, *IL‐6*, *p21*, *p16*, *MCP‐1*) and intestinal barrier‐related proteins (*Tricellulin*, *Occludin*, *Claudin1*, *Claudin4*). Compared with young mice, the expression levels of SASP‐related genes in aged mice were significantly increased, including *Il‐1β*, *TNF‐α*, and *p16*, and the expression levels of intestinal barrier‐related markers *Tricellulin* and *Occludin* were significantly decreased (Figure 1A, Table S3, *p* < 0.05). The proportion of Th cell subsets in the intestinal lamina propria was analyzed by flow cytometry. Naïve Th cells were significantly reduced in the aged intestine (Figure 1B, Figure S1A, *p* < 0.01), while Effector Th cells were significantly increased (Figure 1B, Figure S1A, *p* < 0.05), and the Th cell subsets shifted toward Effector T cells. At the same time, Th cells showed a shift toward Th1 cells and Th17 cells (Figure 1C, Figure S1B–E, *p* < 0.05), suggesting that a chronic inflammatory state associated with aging may have occurred. IgA secretion was also evaluated as an important phenotype of the function of lamina propria immune cells. The content of IgA in the feces of aged mice was significantly reduced (Figure 1D, *p* < 0.001), and there was no significant difference in the serum (Figure 1E, *p* > 0.05). There was no statistically significant difference in fecal LPS content between the two groups (Figure 2A, *p* > 0.05). Serum LPS levels were significantly elevated in aged mice compared to young mice (Figure 2B,p < 0.001). It may be due to the disorder of intestinal mucosal immune function that pathogens and their virulence factors in the aged intestine can penetrate the intestinal barrier and enter the peripheral blood circulation. And the number of Peyer's patch in the aged intestine decreased slightly, but the difference was not statistically significant (Figure 2C), and the M cells with important immune functions in Peyer's patch also underwent aging‐related changes. The expression of M cell differentiation‐related genes such as *Gp2*, and M cell antigen recognition function‐related genes such as *Sox8*, *Ccl20*, *Siglec5* and *Rankl* were significantly reduced (Figure 2D,p < 0.05), which may be the reason for the increase in serum LPS content.

### 3.2 Dysregulated Expression of FAE Genes in the Intestine of Aged Mice

**FIGURE 3:**
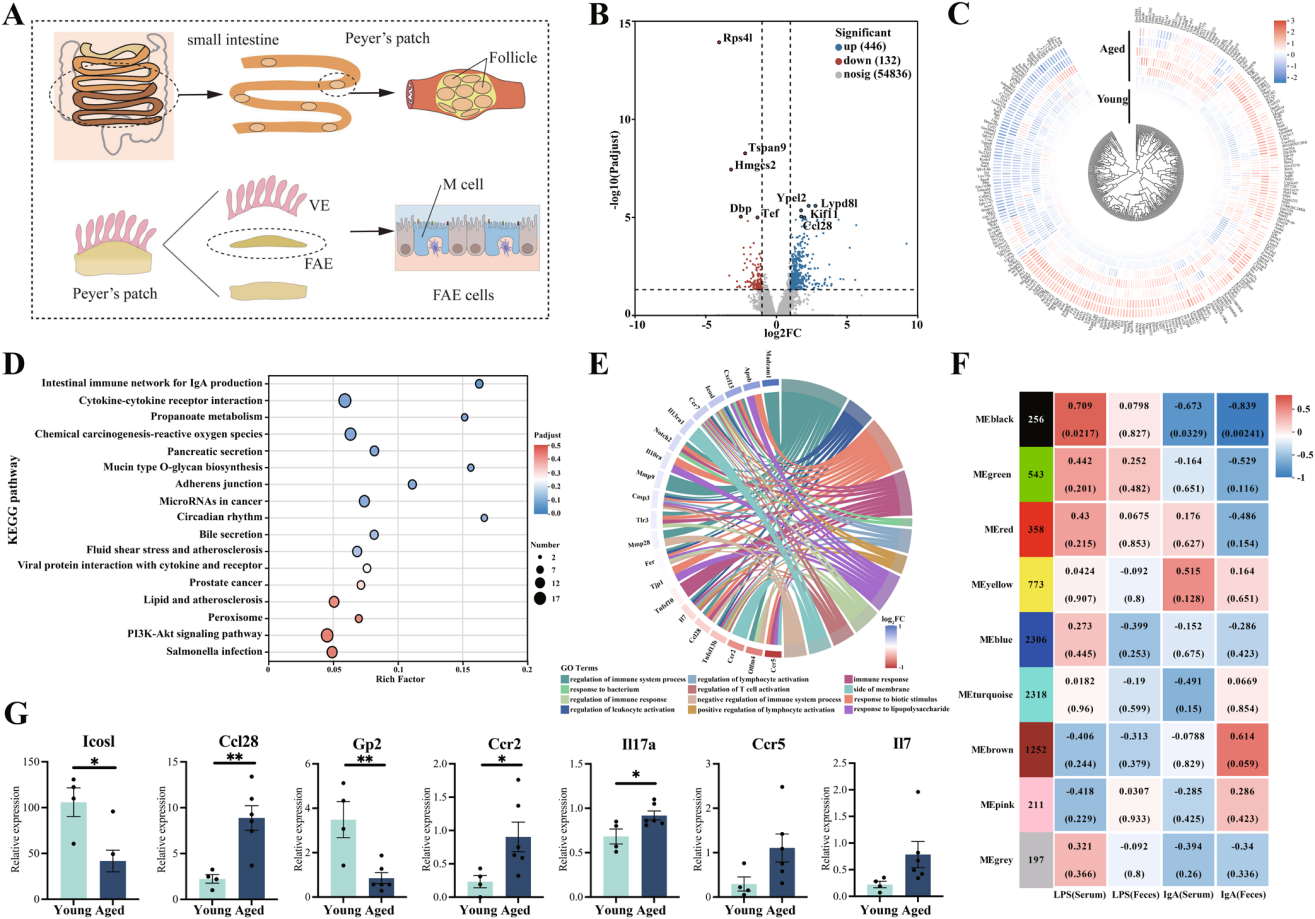
FAE cells exhibited aging‐related gene expression profiles similar to those of M cells. (A) Flowchart for isolating FAE cells from the small intestine of young and aged mice. (B) Volcano plot of differentially expressed genes between young and aged group. (C) Heatmap of differentially expressed genes between young and aged group. (D) KEGG pathway enrichment bubble map. (E) GO pathway enrichment chord diagram. F. Heatmap of correlation between LPS, IgA content and differentially expressed gene modules. G. Expression level of key differential gene (*Icosl*, *Ccl28*, *Gp2*, *Ccr2*, *Il17a*, *Ccr5*, *Il7*). Differential expression analysis was performed using the DESeq2. DEGs with |log_2_FC| ≥ 1 and FDR ≤ 0.05 were considered to be significantly different expressed genes. *p < 0.05, **p < 0.01, ***p < 0.001; two‐tailed Student's *t*‐test.

To elucidate the impact of reduced M cell number and function on intestinal mucosal immunity, we isolated FAE cells—the interface between M cells and the intestinal lumen—from young and aged mice and performed RNA sequencing (Figure 3A). Compared to young mice, FAE cells from aged mice exhibited 578 differentially expressed genes (DEGs), with 446 significantly upregulated and 132 significantly downregulated (Figure 3B,C). These DEGs were enriched in several intestinal mucosal immunity‐related KEGG pathways, including intestinal immune network for IgA production, cytokine‐cytokine receptor interaction, and propanoate metabolism (Figure 3D). Moreover, key DEGs such as *Olfm4*, *Ccr2*, *Il7*, *Ccl28*, and *Ccr5* were also significantly enriched in multiple GO terms related to mucosal immunity, including regulation of immune system process, response to bacterium, regulation of lymphocyte activation, etc. (Figure 3E). Further WGCNA, integrating serum and fecal LPS and IgA levels, clustered all genes into nine modules (Figure 3F). Notably, the black module showed a strong correlation with both LPS and IgA levels. To identify the most biologically significant candidate genes, we found intersections between 256 genes in the black module and gene sets in the significantly enriched KEGG and GO pathways. By combining functional enrichment and WGCNA analyses, we identified seven key DEGs associated with intestinal mucosal immune function: *Icosl*, *Ccl28*, *Gp2*, *Ccr2*, *Il17a*, *Ccr5*, and *Il7* (Figure 3G, Figure S2). *Gp2* was also found to be significantly reduced in patients with inflammatory bowel disease (ulcerative colitis and Crohn's disease) (Figure S5).

### 3.3 Imbalance of Intestinal Microbiome in Aging Mice

**FIGURE 4:**
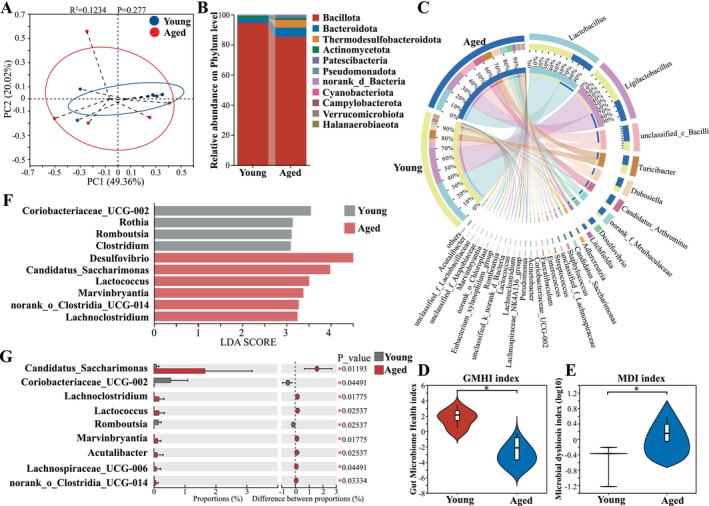
Aging‐associated alterations in the intestinal microbiota. (A) Results of the principal coordinate analysis (PCoA) of young and aged groups based on Bray–curtis dissimilarity. (B) Bar plot of microbial composition at the phylum level. (C) Circos diagram of composition at the genus level. (D, E) Gut microbiome health index (GMHI) and microbial dysbiosis index (MDI), using the young group as the baseline. (F) Linear discriminant analysis (LDA) plot highlighting bacterial genera with significant differences (LDA > 2, *p* < 0.05). (G) Bar plot comparing bacterial taxa between groups based on the Wilcoxon rank‐sum test (*p* < 0.05).

An average of 833,452 high‐quality sequences were obtained after 16S rRNA sequencing screening. Principal coordinate analysis (PCoA) was used to evaluate the similarity of microbial profiles. As shown in Figure 4A, the confidence ellipses of the aged and young groups exhibited substantial overlap. Statistical testing via PERMANOVA (R^2^ = 0.1234, *p* = 0.277) confirmed that there was no significant difference in the global microbial community structure between the two groups (*p* > 0.05), indicating that the core microbiome architecture remains relatively stable during aging. Alpha diversity analysis (Figure S3) revealed that compared to the young group, the aging group showed a significantly higher Chao index (*p* < 0.05) and a significantly lower Simpson index (*p* < 0.05). This indicated that the species richness of the aged group community increased. Compared with the microbial composition of young mice, the proportion of Bacillota decreased and the proportion of Bacteroidota increased in the aged group (Figure 4B). Notably, there was also a visible expansion of the phylum Thermodesulfobacteriota in the aged mice. At the genus level (Figure 4C), the *Lactobacillus* was the most abundant, followed by *Ligilactobacillus*, *unclassified_c_Bacilli*, *Turicibacter*, *Dubosiella*, *Candidatus_Arthromitus*, and *norank_f_ Muribaculaceae*. The abundance of *unclassified_c_Bacilli*, *Dubosiella Candidatus_Arthromitus* and *norank_f_ Muribaculaceae* were higher in the old group, while *Lactobacillus* and *Ligilactobacillus* were less abundance. Furthermore, consistent with the expansion of the Thermodesulfobacteriota, we observed a concurrent increase in the abundance of the *Desulfovibrio* in the aged group. Bacterial typing analysis of the intestinal microbial composition of the two groups found that the gut microbiome health index (GMHI) of the aging group was significantly reduced and the microbial disturbance index (MDI) was significantly increased (Figure 4D,E,p < 0.05). In addition, these bacterial groups were used as biomarkers to distinguish the microbiomes of each group in LEfSe analysis (Figure 4F). As shown in Figure 4F, the microbiomes of the old group were enriched in *Desulfovibrio*, *Candidatus_Saccharimonas*, *Lactococcus*, *Marvinbryantia*, *norank_o_Clostridia_UCG‐014* and *Lachnoclostridium*. Comparative analysis based on the Wilcoxon rank‐sum test also found that the abundance of *Candidatus_Saccharimonas* in the aging group increased significantly (Figure 4G, LDA > 2, *p* < 0.05).

### 3.4 Key Pathogens Highly Correlated With Intestinal Mucosal Immune Dysfunction

**FIGURE 5:**
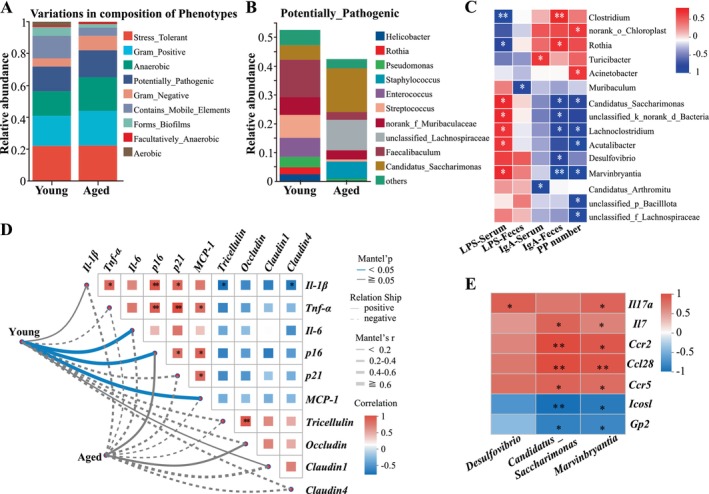
Predictive functional profiling of the gut microbiota and its correlations with mucosal immune features and key differentially expressed genes. (A) Bar plot showing the relative abundance of BugBase‐predicted phenotypic categories in the gut microbiota of young and aged mice. These phenotypic categories are not mutually exclusive, meaning that a single gut microbe in a sample can contribute to multiple predicted phenotypes simultaneously. (B) Bar plot of the relative abundance of species at different genus levels to the “Potentially Pathogenic” phenotypic subset. (C) Spearman correlation heatmap between the relative abundance of differential microbes and intestinal immune phenotypes (LPS levels, IgA content, and Peyer's patch number). (D) Mantel test illustrating the correlation between the overall intestinal microbial community composition (based on Bray‐Curtis distance) and the expression profiles of genes related to intestinal aging and barrier function (based on Euclidean distance). (E) Spearman correlation heatmap between the relative abundance of specific key genera and the expression levels of key differentially expressed genes. *0.01 < *p* ≤ 0.05, **0.001 < *p* ≤ 0.01.

Further BugBase phenotypic prediction analysis of intestinal microbial composition identified nine major phenotypes, including Stress Tolerant, Gram Positive, Anaerobic, Potentially Pathogenic, Gram Negative, Mobile Element Containing, Biofilm Forming, Facultatively Anaerobic and Aerobic. As depicted in Figure 5A, which illustrates the relative proportion of each predicted phenotype across the entire microbial community, the contribution of four phenotypes‐Gram Positive, Anaerobic, Potentially Pathogenic and Gram Negative, increased in the aging group. To further dissect these functional shifts, we performed a targeted taxonomic breakdown of the “Potentially Pathogenic” category (Figure 5B, Figure S4). It is important to note the difference in analytical scope, while the “Potentially Pathogenic” trait constituted approximately 20% of the overall community profile (Figure 5A), Figure 5B specifically details the relative abundance of individual species within this isolated phenotypic subset. This targeted analysis revealed the specific taxonomic drivers (accounting for 40%–55% of the abundance within this subset) responsible for the age‐associated increase in pathogenic potential. Further focusing on the top 10 species contributing to Potentially Pathogenic at the genus level, *Candidatus_Saccharimonas*, which had significant composition differences, increased its contribution by nearly 2 times in the old group. At the same time, in the correlation analysis between LPS and IgA content (Figure 5C), it was found that the content of multiple pathogens, such as *Candidatus_Saccharimonas*, *Desulfovibrio* and *Marvinbryantia* were positively correlated with the content of LPS and negatively correlated with the content of IgA. To further investigate the specific microbial drivers of mucosal immune dysregulation, we narrowed our focus to *Candidatus_Saccharimonas*, *Desulfovibrio* and *Marvinbryantia*. Their significant abundance differences between the groups (Figure 4E,F), and strong correlations with key aging gut phenotypes (such as LPS levels, IgA content, and Peyer's patch numbers; Figure 5C), and their functional classification as potentially pathogenic taxa according to the BugSigDB annotations. Mantel test analysis was performed to evaluate the associations between gut microbiota composition and the expression of aging‐ and barrier‐related genes (Figure 5D). This analysis evaluated the correlation between the overall microbial community structure (based on Bray‐Curtis distance) and the expression profiles of gene sets related to intestinal aging and barrier function (based on Euclidean distance). Mantel analysis revealed a strong association between microbial community structure and *IL‐6*, *p16*, and *MCP‐1* expression in young mice (*r* > 0.6, *p* < 0.05). In contrast, these associations were markedly weaker in aged mice (*r* = 0.2–0.4), despite significantly elevated expression levels of these genes. The heatmap further revealed that aging‐associated genes were positively correlated with each other, whereas barrier integrity genes showed coordinated negative associations with inflammatory and senescence markers. This suggests an age‐related disruption of host‐microbiota coordination rather than a simple enhancement of inflammation‐driven coupling. Combined with the significant changes in relative abundance and phenotypic association analysis, the three key pathogens *Candidatus_Saccharimonas*, *Desulfovibrio* and *Marvinbryantia* may have an important negative impact on the immune function of the intestinal mucosa, and highly correlated with the differential genes with significant changes in aging FAE, with significant positive correlations with *Il17a*, *Il7*, *Ccr2*, *Ccl28*, *Ccr5*, and significant negative correlations with *Icosl* and *Gp2* (Figure 5E).

### 3.5 Aged Mice Were More Susceptible to *Clostridium difficile* Infection

**FIGURE 6:**
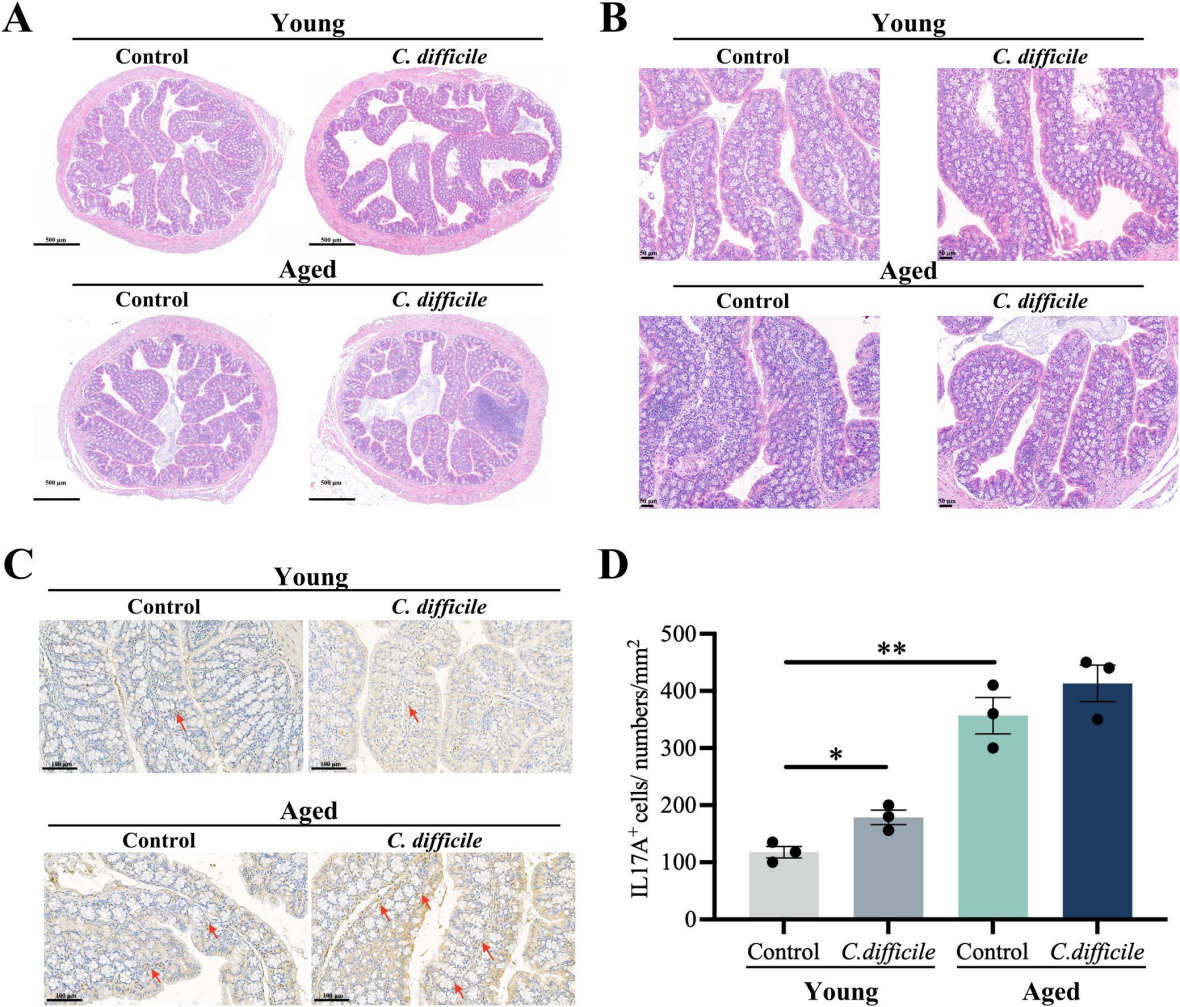
Highly elevated IL‐17A expression in the intestine following *Clostridium difficile* infection. (A, B) Hematoxylin and eosin staining of the colon from young and aged mice infected with *Clostridium difficile* (A: scale bar, 500 μm; B: Scale bar, 50 μm). (C, D) Representative images and quantification of IL‐17A immunohistochemical staining in the colon of infected mice. *p < 0.05, **p < 0.01; two‐tailed Student's *t*‐test.

We further investigated immune dysregulation in the aged intestinal mucosa using a *Clostridium difficile* infection model (Figure 6A). Following infection, the intestines of aged mice displayed more severe inflammatory responses, and obvious inflammatory infiltration was observed in HE‐stained sections (Figure 6A,B). Immunohistochemical analysis confirmed that the number of IL‐17A‐positive cells was significantly increased in aged mice (Figure 6C,D). Notably, even in the absence of infection, aged mice showed higher IL‐17A levels compared to young mice, whereas only the young group exhibited a significant increase in IL‐17A positivity after infection (Figure 6D).

## 4 Discussion

This study systematically investigated the multifaceted effects of aging on the intestinal mucosal immune system in mice, with a particular focus on age‐related alterations in microbial composition and the regulation of the M cell gene expression network, ultimately contributing to increased susceptibility to intestinal pathogens. The findings suggested that a potential mechanism involves the expansion of key pathogenic bacteria, such as *Candidatus_Saccharimonas* and *Desulfovibrio*, in the aged intestine, which aggravated pro‐inflammatory changes in intestinal immune cells. This, in turn, led to dysregulation of genes associated with M cell‐mediated antigen recognition and presentation. These results may offer novel targets for modulating intestinal immune function in the elderly. First, We observed a significant upregulation of key senescence‐associated secretory phenotype (SASP) factors, including *IL‐1β*, *TNF‐α*, and *p16*, accompanied by a marked reduction in tight junction proteins *Tricellulin* and *Occludin* in the aged intestine. This was consistent with the concept of “inflammaging” characterized by chronic, systemic low‐grade inflammation that accompanies aging and predisposes to various diseases (Franceschi et al. 2018). The disruption of intestinal tight junction compromises barrier integrity, increasing the translocation of microbial products such as LPS into circulation (Ghosh et al. 2020). This was also confirmed by existing findings that aged mice show increased intestinal permeability, systemic inflammation, and altered macrophage function (Di Vincenzo et al. 2024). Additionally, flow cytometric analysis revealed a shift in T helper (Th) cell populations within the intestinal lamina propria. Aging led to a reduction in naïve Th cells and an increase in effector Th cells, with a skewing toward Th1 and Th17 subsets (Figure 1). In various inflammatory bowel disease (IBD) models, intestinal CD4^+^ T cells also preferentially differentiated into Th1 cells, and in some Crohn's disease (CD) patients, IL‐23‐ and IL‐17‐producing cells were predominant in the lesions (Saurer and Mueller 2009). These findings suggested that intestinal T cells in naturally aged individuals exhibited characteristics similar to those observed in IBD. Among these subsets, Th17 cells recruited and activated immune cells such as neutrophils by secreting cytokines like IL‐17, which were essential for maintaining mucosal immunity (Yan et al. 2020). But excessive Th17 responses could lead to severe inflammation and promote the development of various autoimmune diseases (Chen et al. 2022). Aged mice exhibited elevated baseline levels of IL‐17A, even prior to infection, highlighting the pro‐inflammatory milieu of the aging intestine. Moreover, the IgA content in the feces of aged mice also decreased significantly (Figure 1D), reflecting the functional decline of B cells in secreting IgA (Lycke and Bemark 2017). When evaluating age‐related changes in the intestinal mucosal immune system, two notable phenomena were observed. First, there was a significant increase in LPS levels in serum (Figure 2B), suggesting that aging may be associated with dysbiosis of the intestinal microbiota (Kim et al. 2016). Second, although the absolute number of Peyer's patches did not decrease significantly, the age‐associated functional impairment of these structures—particularly the decline in M cells, which are responsible for recognizing and transporting pathogens—may contribute to increased susceptibility to intestinal diseases (Kobayashi et al. 2019).

To further clarify the changes in the function of M cells in Peyer's patches in aging mice, we performed transcriptome analysis on follicle‐associated epithelial (FAE) cells, revealing 578 differentially expressed genes (DEGs) in aged mice, many of which were enriched in pathways critical for mucosal immunity, including the “Intestinal immune network for IgA production” and “Cytokine‐cytokine receptor interactions”. Notably, genes such as *Gp2*, *Sox4*, *Ccl28*, *Ccr2*, *Il17a*, and *Ccr5* were prominently altered. Gp2 is a well‐established marker of M cells that mediates bacterial transcytosis, facilitating antigen sampling and delivery to Peyer's patches (Mabbott et al. 2013). The downregulation of *Gp2* and other M cell‐associated genes suggests impaired antigen sampling, which may diminish the initiation of mucosal immune responses. Similarly, reduced expression of Ccl28, a chemokine crucial for IgA plasma cell homing (Rios et al. 2016), could explain the decreased fecal IgA levels observed in aged mice. WGCNA further identified key modules correlating with fecal and serum LPS and IgA concentrations. Genes within these modules, including *Icosl*, *Ccl28*, *Gp2*, *and Il17a*, appear to form a regulatory nexus linking mucosal gene expression with systemic indicators of barrier and immune dysfunction. This highlights how aging‐driven transcriptional changes in FAE cells may orchestrate broader immune alterations. However, the results of FAE cells in this study are mainly based on transcriptomics and association analysis, and further functional verification is still needed.

The enrichment of LPS in the feces and serum of aged mice indicated age‐associated alterations in the intestinal microbiota. As a critical component of the intestinal mucosal immune system, the gut microbiota can directly or indirectly modulate immune function (Bosco and Noti 2021). 16S rRNA sequencing demonstrated profound shifts in the gut microbial composition of aged mice. Subtle changes in diversity indices (Shannon, Chao, and Simpson index) and gut microbiota composition suggest that the impact of aging on gut microbiota diversity may be more complex. While aging has traditionally been associated with a gradual loss of microbiota diversity (Langille et al. 2014), recent high‐resolution studies have revealed a more nuanced picture. A comprehensive meta‐analysis of the mouse microbiome showed that aging increases alpha diversity in the gut microbiota (You et al. 2022). The large cohorts of adults have also found a significant positive correlation between age and alpha diversity in young adults (de la Cuesta‐Zuluaga et al. 2019). Conversely, a large‐scale human cohort study showed that aging does not affect alpha diversity, but rather exhibits an increasingly unique microbiota composition trajectory that predicts survival (Wilmanski et al. 2021). These findings suggest that the impact of aging on microbial diversity cannot be generalized in isolation and must be considered in conjunction with changes in specific community structures. We observed decreased proportions of Bacillota (formerly Firmicutes), alongside increased Bacteroidota and enrichment of potentially pathogenic genera such as *Candidatus_Saccharimonas*, *Desulfovibrio*, and *Marvinbryantia*. *Candidatus_Saccharimonas* was an important regulatory gene and key differential bacteria in the onset of intestinal adenoma (Guo et al. 2021). Study focusing on liver and fat inflammation also found that *Candidatus_Saccharimonas* was significantly enriched (Yan et al. 2024). *Desulfovibrio* was a sulfate‐reducing bacterium known to produce hydrogen sulfide, which can impair epithelial barrier function and promote inflammation (Singh et al. 2023). Intestinal inflammation was alleviated after inhibiting *Marvinbryantia* and related niacin and nicotinamide metabolite pathways (Niu et al. 2024). However, the biological role of *Marvinbryantia* is highly complex and context‐dependent. As a member of the Lachnospiraceae family, it has been reported to be strongly negatively correlated with cirrhosis risk (Yuan et al. 2024), and significantly reduced in elderly cirrhotic patients with low muscle mass (Han et al. 2022), suggesting a potential protective role, possibly through short‐chain fatty acid (SCFA) production. The paradoxical expansion of *Marvinbryantia* in our aging model suggests that its function may shift from beneficial to detrimental under the specific microenvironmental pressures of the aging gut, or that its overgrowth is a compensatory response to age‐associated mucosal damage. Notably, this potential association with *Marvinbryantia* is based primarily on predictive functional annotations rather than direct experimental verification, and therefore should be interpreted with caution. Direct functional and mechanistic validation is needed to determine whether these specific taxa actively drive this phenotype. It is noteworthy that the relationship between the gut microbiota and the host is highly dynamic and bidirectional, especially during aging (Bosco and Noti 2021). Our findings strongly confirm the close association between three significantly enriched potential pathogens and epithelial gene expression dysregulation and decreased mucosal immunity. However, intrinsic host aging factors may also be major drivers of these microbial community changes (Gao et al. 2026). With increasing host age, physiological changes in the gastrointestinal tract, including decreased intestinal motility and altered gastric acid secretion, create a favorable environment for the overgrowth of pathogenic bacteria. Furthermore, the degradation of the intestinal mucosal microenvironment—characterized by a thinning mucus layer, reduced mucin production, and decreased secretion of antimicrobial substances, also contributes to the overgrowth of pathogenic bacteria. Peptides exert profound selective pressure, reshaping the microbial community structure (Sovran et al. 2019). Simultaneously, immunosenescence (age‐related decline in immune system function) weakens the host's ability to maintain immune surveillance of the microbiota, further exacerbating dysbiosis. Therefore, the gut immune dysfunction observed in aged mice is likely the result of a complex, interdependent feedback loop, rather than a one‐way microbial effect. Future research needs to incorporate mechanistic models to fully elucidate the causal orientation of host‐microbiome interactions during gut aging.

Furthermore, this study focused on aging‐related alterations in intestinal immune function—such as changes in LPS, IgA levels, and Peyer's patch number‐as well as the association between intestinal microbiota imbalance and the FAE gene expression network. Consistent with previous studies, it is well documented that aged mice exhibited a marked decline in intestinal mucosal immune function, characterized by a significant thinning of the mucus layer and epithelial barrier defects (Goepp et al. 2025), including reduced secretion of mucin and lysozyme by goblet cells and Paneth cells in the intestinal crypts and villi (Hohman and Osborne 2022). Previous research has also demonstrated that these changes were accompanied by major shifts in the fecal microbiota, notably a significant reduction in the abundance of *Akkermansia muciniphila* (Sovran et al. 2019). Building upon this context, our current transcriptomic analysis of intestinal mucosal tissue revealed age‐associated downregulation of immune‐related genes, including those involved in T cell‐specific functions and signaling pathways. Our data suggest that changes in intestinal microbial composition and the decline in intestinal immune cell function may not only occur simultaneously but also interact with each other. In particular, we observed that aging‐induced shifts in specific microbial taxa and the expansion of pro‐inflammatory pathogens were strongly associated with the dysregulation of genes involved in M cell‐mediated antigen recognition, which may contribute to microbial translocation and disrupting immune cell homeostasis. Supporting this microbe‐host interaction, other studies have verified that *Segmented Filamentous Bacteria* (SFB) promoted the production of Th17 cell cytokines, and mice treatment with SFB flagellin led to a significant increase in the expression of genes related to the IL‐17 signaling pathway and affected the expression of epithelial cell‐specific genes, such as *Nos2* and *Duox2* (Wang et al. 2019). In our present study, the functional consequences of these aging‐associated changes were strikingly illustrated in our *Clostridium difficile* infection model. We found that aged mice exhibited more severe histopathological damage, characterized by pronounced inflammatory infiltration and elevated IL‐17A expression. Our findings align with clinical observations that elderly patients are at higher risk of *Clostridium difficile* infection and recurrence, partly due to age‐related declines in mucosal immunity and microbiome resilience (Buffie et al. 2015). Interestingly, the literature indicates that IL‐17A plays a dual role in *C. difficile* infections. While it can promote neutrophil recruitment and pathogen clearance, excessive IL‐17A responses may exacerbate tissue damage (Saleh et al. 2019). Based on our results, we hypothesize that the already elevated baseline IL‐17A in aged mice likely predisposes them to an exaggerated inflammatory response upon infection, contributing to worse outcomes. This pathway can provide new interventions for aging intestinal mucosal immune dysfunction. But when interpreting the *C. difficile* infection data, species‐specific differences in bile acid metabolism should be considered. Primary bile acids such as taurocholate promote *C. difficile* spore germination, whereas secondary bile acids, including deoxycholate and lithocholate, inhibit germination and growth (Sorg and Sonenshein 2008). Mice have a bile acid pool enriched in taurine‐conjugated bile acids and muricholic acids, while humans exhibit higher levels of glycine‐conjugated and secondary bile acids (Collins et al. 2023; Sayin et al. 2013). These differences are further shaped by microbiota‐dependent bile acid transformations, which are disrupted by antibiotics and directly influence susceptibility to *C. difficile* infection (Theriot et al. 2014). Therefore, murine infection outcomes may not fully reflect human disease dynamics. Drawing from existing literature, microbiome‐targeted therapies, including specific probiotics or fecal microbiome transplantation, can restore beneficial bacteria such as *Lactobacillus* and inhibit opportunistic pathogens (Hitch et al. 2022). Additionally, other promising strategies include promoting M cell differentiation through factors such as RANKL, thereby promoting antigen acquisition and IgA production (Knoop et al. 2009). And regulate the IL‐17/CCR5 pathway to balance protective immunity and chronic inflammation risk (Sun et al. 2023). Enhancing tight junction integrity using methods such as NAD^+^ precursors has also been shown to reduce SASP and improve epithelial barrier function in aged mice (Zhang et al. 2016).

Although the elderly SPF C57BL/6 mouse model in this study provides valuable mechanistic inferences for elucidating age‐related mucosal changes, these findings have certain limitations when applied to the elderly due to physiological differences between humans and mice. First, standard SPF mice reside in highly controlled, ultra‐clean environments, resulting in relatively naive, low‐exposure immune systems even in old age. In contrast, adult humans accumulate lifelong complex environmental, microbial, and pathogenic exposures that continuously shape mucosal immune memory and chronic inflammatory tones (Beura et al. 2016). Second, the murine and human gut microbial communities are compositionally distinct. Several microbial taxa that dominate the mouse gut ecosystem—and which may play central roles in our described phenotypes—do not map cleanly onto the adult human microbiome (Hugenholtz and de Vos 2018). Third, significant species‐specific differences exist in mucosal immune organization and effector programs. For instance, the robust induction of Th17 differentiation and specific IgA responses in the murine gut is frequently driven by murine‐specific commensals (such as Segmented Filamentous Bacteria), whose functional equivalents in humans either remain elusive or operate through divergent pathways (Honda and Littman 2016; Mestas and Hughes 2004). Therefore, while the overarching concept of age‐related FAE transcriptional remodeling may be evolutionarily conserved, the specific microbial drivers and mucosal effector phenotypes observed in our study are largely mouse‐context specific. In the future, mechanism validation using human organoids or appropriately exposed matching transformational models will be crucial to bridging this gap. Currently, most available human ileal transcriptome datasets are derived from pathological conditions, such as inflammatory bowel disease, whereas targeted biopsy datasets from healthy elderly individuals remain scarce. In addition, M cells and FAE cells constitute only a very small proportion (< 1%) of intestinal epithelial cells and are therefore likely to be substantially diluted in bulk RNA sequencing datasets from ileal biopsies, further limiting their detectability. Collectively, these scarcity of clinical studies and sample sites also provide direction for future research. With aging, atrophy of gut‐associated lymphoid tissue, impaired germinal center responses, and reduced antigen sampling efficiency are commonly observed in mammals. The M cell differentiation features discussed in this study may be highly conserved between humans and mice, particularly with respect to RANKL expression levels. Existing studies have demonstrated that the RANKL‐RANK signaling axis serves as a central mechanism driving FAE differentiation in both species (Akiyama et al. 2012). Similarly, microbiota‐derived regulation of epithelial barrier function, including effects mediated by short‐chain fatty acids, appears to be evolutionarily conserved. However, the composition and complexity of the gut microbiota differ substantially between humans and mice. Moreover, the SPF housing conditions of laboratory mice result in markedly reduced immune stimulation compared with humans. From a physiological perspective, differences also exist between species in the number and distribution of Peyer's patches in the ileum, as well as in the transcriptomic profiles of FAE cells. And future causal validation should employ complementary in vivo models, including colonization of germ‐free (GF) mice with defined microbial consortia or transplantation of key microorganisms into GF or antibiotic‐treated mice. Germ‐free mice provide a powerful reductionist system for dissecting host–microbiota interactions; however, they exhibit marked developmental abnormalities in gut‐associated lymphoid tissues (GALT), including reduced Peyer's patches, underdeveloped lymphoid structures, and decreased lamina propria immune cell populations, which may exaggerate mucosal phenotypes and alter immune responsiveness (Round and Mazmanian 2009). These immune immaturities reflect the essential role of microbial colonization in postnatal development of mucosal immunity (Hooper and Macpherson 2010). In contrast, human microbiota‐associated (HMA) mice partially recapitulate human microbial composition but fail to fully maintain donor community structure over time, with colonization bias toward murine‐adapted taxa and reduced representation of strict anaerobes (Gerner et al. 2025). As a result, HMA models may improve translational relevance but still diverge substantially from the original human donor microbiome. Therefore, robust causal inference likely requires a combination of complementary systems, including antibiotic‐cleared conventional mice, GF mice with defined microbial reconstitution, and more ecologically relevant “wildling” or rewilded mouse models, which better recapitulate natural microbial exposure and immune maturation (Buffie et al. 2015). Integrating these models may provide a more physiologically representative framework for dissecting host–microbiota interactions in mucosal immunity.

In summary, this study highlights that during the aging process, the intestinal microbiota exhibited alterations in specific bacterial populations. The proliferation of specific potentially pathogenic genera, such as *Candidatus_Saccharimonas*, *Desulfovibrio*, and *Marvinbryantia*, is strongly associated with the dysregulation of key genes related to M cell antigen recognition (*Gp2*, *Ccr2*, *Ccl28*, *Ccr5*, etc.). Furthermore, these microbial shifts correlate with increased microbial translocation, a shift toward pro‐inflammatory mucosal immune cell populations, and an overall decline in intestinal immune function. Importantly, we acknowledge that the relationship between the gut microbiota and the host is highly dynamic and bidirectional. While our findings suggest that the expansion of these key pathogens may contribute to immune dysregulation, it is equally plausible that host‐intrinsic aging factors, such as immunosenescence and degraded mucosal barriers, act as primary drivers that reshape the gut microbial community. Because our current study relies on cross‐sectional correlations, it cannot definitively establish the causal direction of these interactions. Nevertheless, the complex interplay among these genetic, cellular, and ecological factors likely underpins the increased susceptibility of the elderly to intestinal infections and inflammatory diseases. By comprehensively analyzing these multi‐level interactions, we can better design targeted interventions to maintain intestinal health and systemic immunity in the aging population.

## Author Contributions

**Fang Wu:** writing – original draft, methodology, data curation, formal analysis. **Ming Zhang:** conceptualization, investigation, writing – review and editing. **Jianmin Wu:** methodology, data curation. **Zihan Wang:** data curation. Yumeng Ma: data curation. **Lin Dong:** data curation. **Le Cheng:** data curation. **Tengteng Ji:** data curation. **Chenyan Zheng:** data curation. **Fazheng Ren:** resources, funding acquisition. **Bing Fang:** conceptualization, funding acquisition, investigation, supervision, writing – review and editing.

## Funding

The work was supported by Beijing Nova Program (20240484606), the Key Project of Henan Science and Technology Research and Development Plan (241110110200), the Cross‐Innovation Open Project of Food Flavor and Health, Beijing Technology & Business University (FFHCI‐2025061), and the 2115 Talent Development Program of China Agricultural University.

## Ethics Statement

All experiments involving animals were conducted according to the ethical policies and procedures, followed National Research Council's Guide for the Care and Use of Laboratory Animals and approved by the Ethics Committee of China Agricultural University (AW40113202‐5‐2).

## Conflicts of Interest

The authors declare no conflicts of interest.

## Supporting information


**Figure S1:** Gating strategy for flow cytometry analysis. (A) Representative flow cytometry plots showing the proportions of Naïve, Effector and Memory Th cells in the intestinal lamina propria. (B–D) Representative flow cytometry plots showing the proportions of Th1 (B), Th2 (C), and Th17 (D) cells in the intestinal lamina propria. (E) Hierarchical gating strategy for the identification of Th17 cells.
**Figure S2:** Identification of candidate genes via intersection analysis. Venn diagrams illustrate the number of shared genes across different gene sets. (A) Intersection between DEGs identified in KEGG enrichment analysis and GO enrichment analysis. (B) Intersection between KEGG‐enriched DEGs and genes identified in the WGCNA black module. (C) Intersection between GO‐enriched DEGs and genes identified in the WGCNA black module.
**Figure S3:** Alterations in microbial alpha diversity during aging. (A–C) Boxplots showing comparisons of the Shannon index (A), Chao index (B), and Simpson index (C) between the young and aged groups. Statistical significance was determined using Student's *t*‐test. *p < 0.05.
**Figure S4:** Relative abundance of species contributing to the phenotypic subsets. (A–H) Bar plot of the relative abundance of species at different genus levels to the “Stress Tolerant” (A), “Aerobic” (B), “Gram Negative” (C), “Gram Positive” (D), “Anaerobic” (E), “Forms Biofilms” (F), “Contains Mobile Element” (G), “Facultatively anaer” (H) phenotypic subset, respectively.
**Figure S5:** Gp2 expression is downregulated in human intestinal inflammatory diseases. (A) Gp2 expression in ileal tissue from patients with Crohn's disease (CD) and healthy controls. (B) Gp2 expression in colonic tissue from patients with ulcerative colitis (UC) and healthy controls. *p < 0.05, **p < 0.01, ***p < 0.001. Data are shown for 54 healthy controls, 43 patients with CD, and 27 patients with UC.
**Table S1:** The list of antibodies used in flow cytometry analysis.
**Table S2:** The list of qPCR primer sequences.
**Table S3:** Expression levels of SASP and barrier markers in the intestinal mucosa of young and aged mice.

## Data Availability

The data that support the findings of this study are available from the corresponding author upon reasonable request.
