# Supplementary material for: Aged Gut Microbiota Induces Mucosal Transcriptional Dysregulation, Impairing Immune Surveillance
Source: Aging Cell. 2026 May 4;25(5):e70533. doi: 10.1111/acel.70533 (PMC13139644; doi:10.1111/acel.70533)
Supplement: Supplementary file 1 — Figure S1: Gating strategy for flow cytometry analysis. (A) Representative flow cytometry plots showing the proportions of Naïve, Effector and Memory Th cells in the intestinal lamina propria. (B–D) Representative flow cytometry plots showing the proportions of Th1 (B), Th2 (C), and Th17 (D) cells in the intestinal lamina propria. (E) Hierarchical gating strategy for the identification of Th17 cells. Figure S2: Identification of candidate genes via intersection analysis. Venn diagrams illustrate the number of shared genes across different gene sets. (A) Intersection between DEGs identified in KEGG enrichment analysis and GO enrichment analysis. (B) Intersection between KEGG‐enriched DEGs and genes identified in the WGCNA black module. (C) Intersection between GO‐enriched DEGs and genes identified in the WGCNA black module. Figure S3: Alterations in microbial alpha diversity during aging. (A–C) Boxplots showing comparisons of the Shannon index (A), Chao index (B), and Simpson index (C) between the young and aged groups. Statistical significance was determined using Student's t‐test. *p < 0.05. Figure S4: Relative abundance of species contributing to the phenotypic subsets. (A–H) Bar plot of the relative abundance of species at different genus levels to the “Stress Tolerant” (A), “Aerobic” (B), “Gram Negative” (C), “Gram Positive” (D), “Anaerobic” (E), “Forms Biofilms” (F), “Contains Mobile Element” (G), “Facultatively anaer” (H) phenotypic subset, respectively. Figure S5: Gp2 expression is downregulated in human intestinal inflammatory diseases. (A) Gp2 expression in ileal tissue from patients with Crohn's disease (CD) and healthy controls. (B) Gp2 expression in colonic tissue from patients with ulcerative colitis (UC) and healthy controls. *p < 0.05, **p < 0.01, ***p < 0.001. Data are shown for 54 healthy controls, 43 patients with CD, and 27 patients with UC. Table S1: The list of antibodies used in flow cytometry analysis. Table S2: The list of qPCR primer sequenc [file ACEL-25-e70533-s001.docx]

**Supplementary information**

**
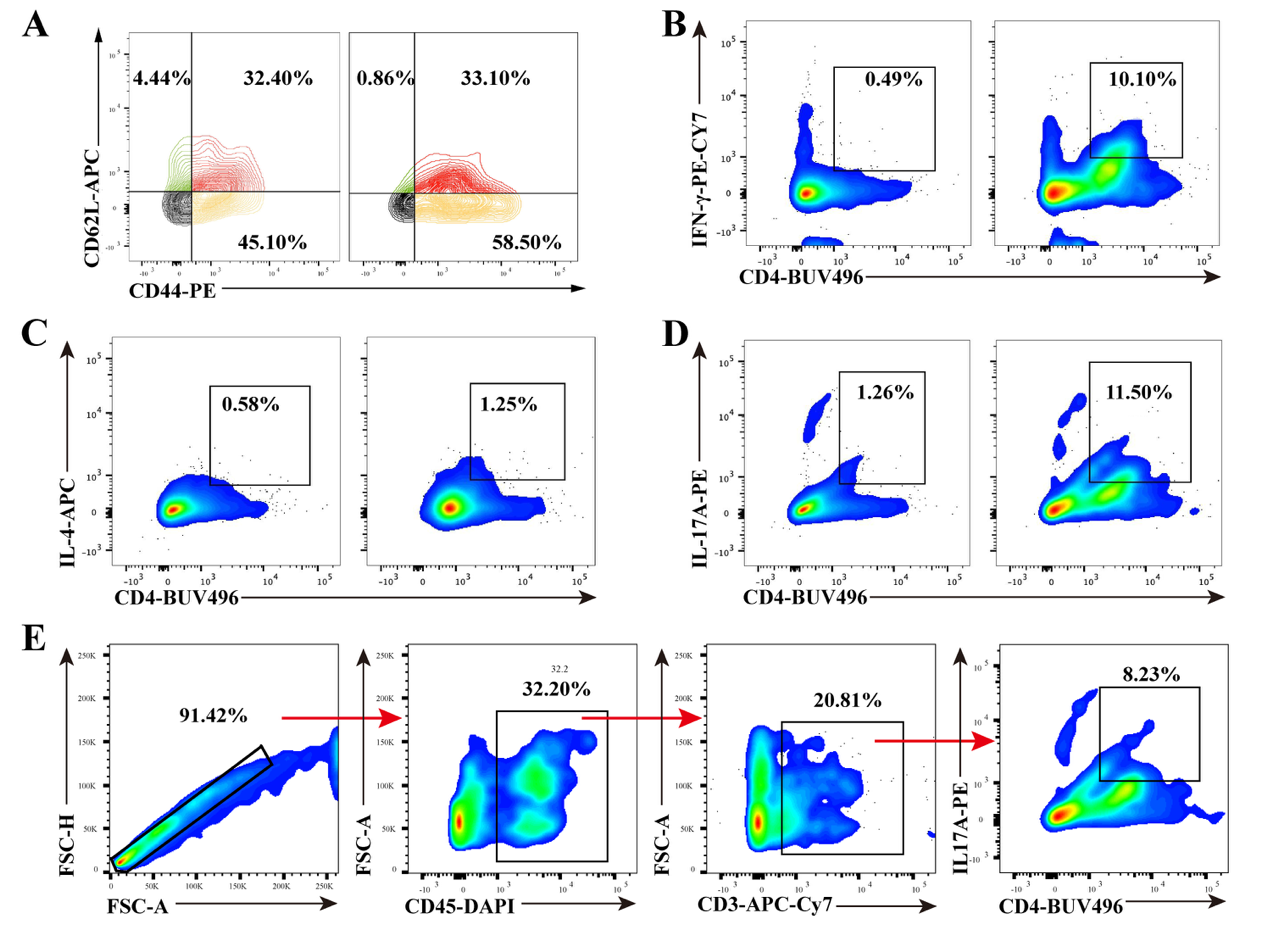
**

**Supplementary Fig. 1. Gating strategy for flow cytometry analysis.** (A) Representative flow cytometry plots showing the proportions of Naïve, Effector and Memory Th cells in the intestinal lamina propria. (B-D) Representative flow cytometry plots showing the proportions of Th1 (B), Th2 (C), and Th17 (D) cells in the intestinal lamina propria. (E) Hierarchical gating strategy for the identification of Th17 cells.


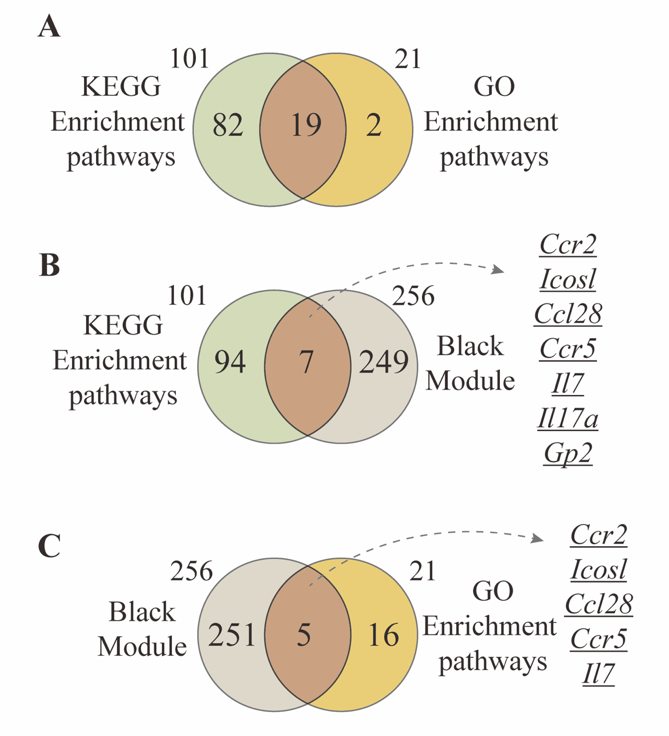


**Supplementary Fig. 2. Identification of candidate genes via intersection analysis.** Venn diagrams illustrate the number of shared genes across different gene sets. (A) Intersection between DEGs identified in KEGG enrichment analysis and GO enrichment analysis. (B) Intersection between KEGG-enriched DEGs and genes identified in the WGCNA black module. (C) Intersection between GO-enriched DEGs and genes identified in the WGCNA black module.

**
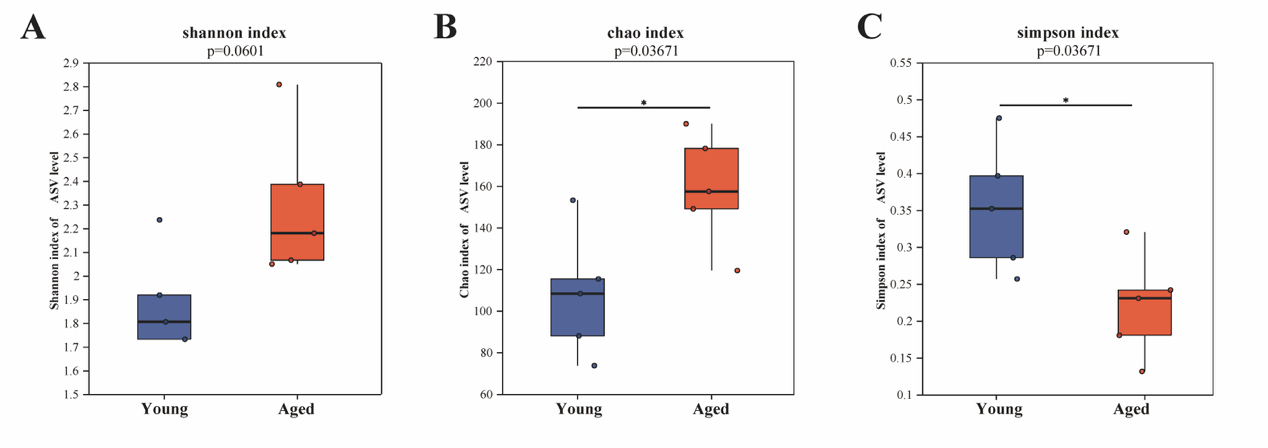
**

**Supplementary Fig. 3. Alterations in microbial alpha diversity during aging.** (A-C) Boxplots showing comparisons of the Shannon index (A), Chao index (B), and Simpson index (C) between the young and aged groups. Statistical significance was determined using Student’s t-test. **p* < 0.05.

**
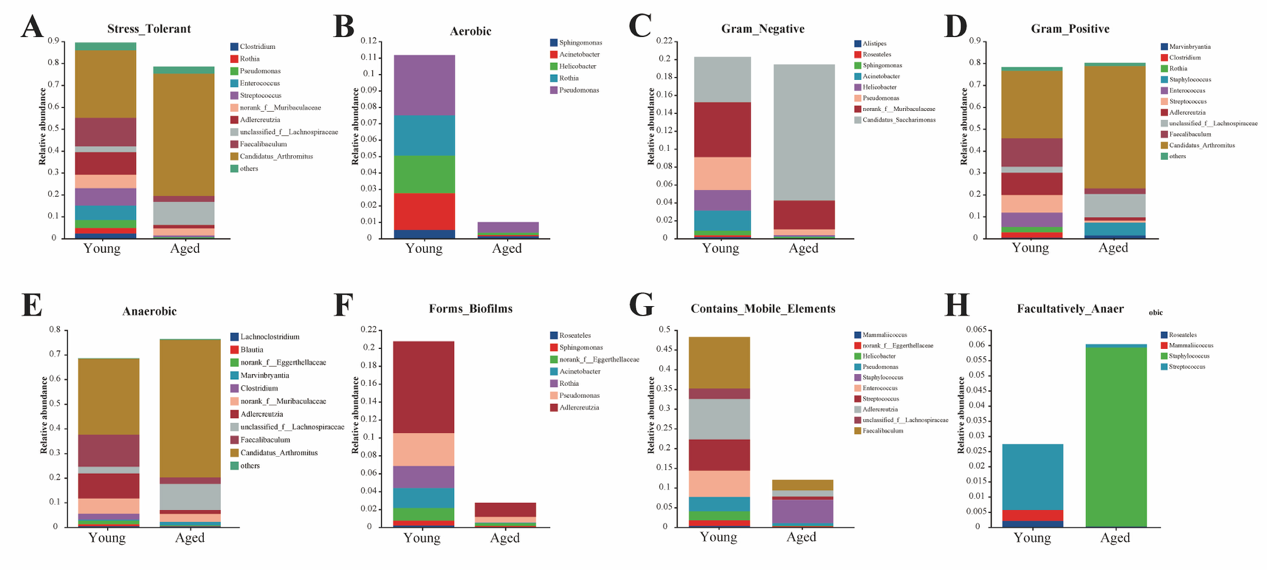
**

**Supplementary Fig. 4. Relative abundance of species contributing to the phenotypic subsets.** (A-H) Bar plot of the relative abundance of species at different genus levels to the “Stress Tolerant” (A), “Aerobic” (B), “Gram Negative” (C), “Gram Positive” (D), “Anaerobic” (E), “Forms Biofilms” (F), “Contains Mobile Element” (G), “Facultatively anaer” (H) phenotypic subset, respectively.

**
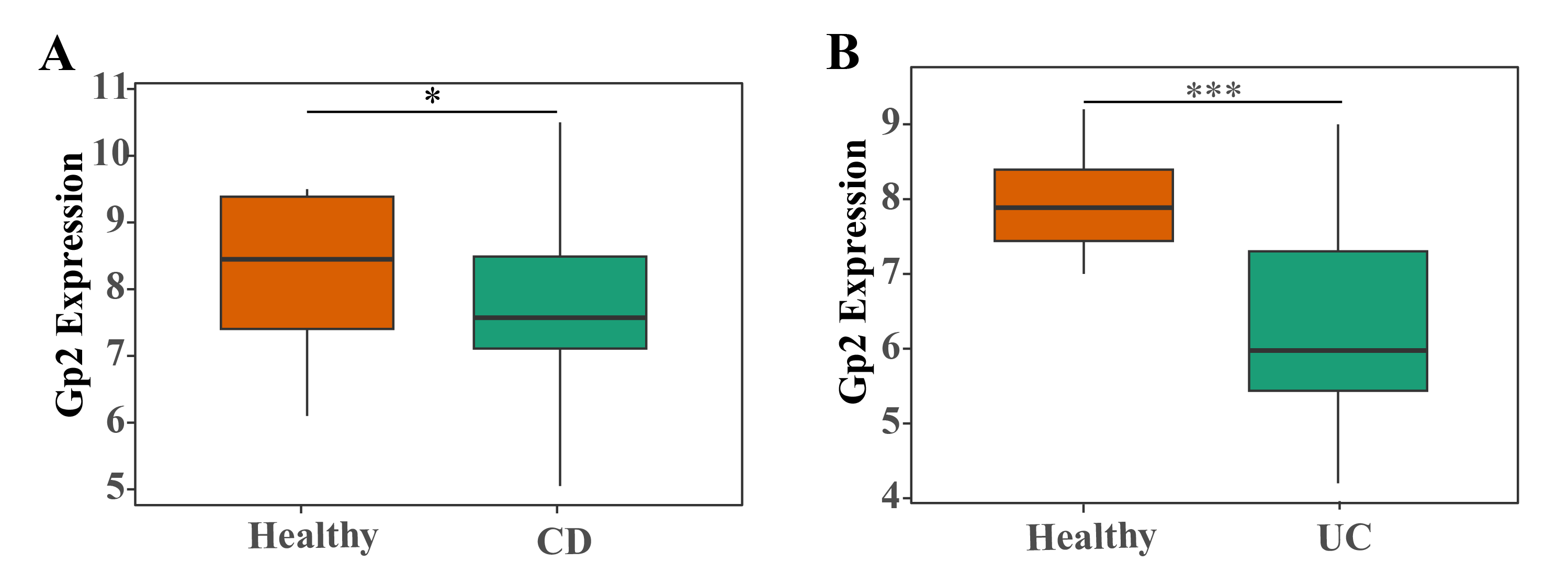
**

**Supplementary Fig. 5. Gp2 expression is downregulated in human intestinal inflammatory diseases.** (A) Gp2 expression in ileal tissue from patients with Crohn’s disease (CD) and healthy controls. (B) Gp2 expression in colonic tissue from patients with ulcerative colitis (UC) and healthy controls. **p* < 0.05, ***p* < 0.01, ****p* < 0.001. Data are shown for 54 healthy controls, 43 patients with CD, and 27 patients with UC.

**Supplementary Table 1 The list of antibodies used in flow cytometry analysis**

| Label | Marker | Clone | Suppliers | Catalog numbers |
| --- | --- | --- | --- | --- |
| Brilliant Violet 421 | CD45 | 30-F11 | Biolegend | 103134 |
| APC/Cyanine7 | CD3 | 17A2 | Biolegend | 100222 |
| PE/Cyanine7 | IFN-γ | XMG1.2 | Biolegend | 505826 |
| APC | IL-4 | 11B11 | Biolegend | 504106 |
| BUV496 | CD4 | RM4-5 | BD | 741050 |
| PE | IL-17A | TC11-18H10 | BD | 559502 |

**Supplementary Table 2 The list of qPCR primer sequences**

| Primer | Primer sequences (5' to 3') |
| --- | --- |
| *IL-1β-F'* | TGGACCTTCCAGGATGAGGACA |
| *IL-1β-R'* | GTTCATCTCGGAGCCTGTAGTG |
| *TNF-α-F'* | GGTGCCTATGTCTCAGCCTCTT |
| *TNF-α-R'* | GCCATAGAACTGATGAGAGGGAG |
| *IL-6-F'* | TACCACTTCACAAGTCGGAGGC |
| *IL-6-R'* | CTGCAAGTGCATCATCGTTGTTC |
| *p16-F'* | TGTTGAGGCTAGAGAGGATCTTG |
| *p16-R'* | CGAATCTGCACCGTAGTTGAGC |
| *p21-F'* | TCGCTGTCTTGCACTCTGGTGT |
| *p21-R'* | CCAATCTGCGCTTGGAGTGATAG |
| *MCP-1-F'* | GCTACAAGAGGATCACCAGCAG |
| *MCP-1-R'* | GTCTGGACCCATTCCTTCTTGG |
| *Tricellulin-F'* | CCACATTCCGAAGCCTATCGTG |
| *Tricellulin -R'* | TGCACTTCCGCAGACAGCTCTT |
| *Occludin-F'* | TGGCAAGCGATCATACCCAGAG |
| *Occludin -R'* | CTGCCTGAAGTCATCCACACTC |
| *Claudin1-F'* | GGACTGTGGATGTCCTGCGTTT |
| *Claudin1-R'* | GCCAATTACCATCAAGGCTCGG |
| *Claudin4-F'* | CGAGCCCTTATGGTCATCAGCA |
| *Claudin4-R'* | ATGCTTGCCACGATGAACACGG |
| *Gp2-F'* | GACATGAGCGTCAGCCTCGAAA |
| *Gp2-R'* | GGAAGCAACACTTCGGCTCCTT |
| *Spib-F'* | AGGAGTCTTCTACGACCTGGAC |
| *Spib-R'* | GGAGTGGCTAAAGGCAGCAGTA |
| *Sox8-F'* | CGCATCTCCATAACGCAGAGCT |
| *Sox8-R'* | TCTTCCTTCGCCTTGGCTGGTA |
| *Ccl20-F'* | GTGGGTTTCACAAGACAGATGGC |
| *Ccl20-R'* | CCAGTTCTGCTTTGGATCAGCG |
| *Siglec5-F'* | CTCCACAGAAGATGACCATCAGG |
| *Siglec5-R'* | CTGTCAGCCATACAGACCAGGC |
| *Rankl-F'* | GTGAAGACACACTACCTGACTCC |
| *Rankl-R'* | GCCACATCCAACCATGAGCCTT |
| *GAPDH-F'* | CATCACTGCCACCCAGAAGACTG |
| *GAPDH-R'* | ATGCCAGTGAGCTTCCCGTTCAG |

**Supplementary Table 3 Expression levels of SASP and barrier markers in the intestinal mucosa of young and aged mice**

| Gene Name | Young Group  (Mean ± SD) | Aged Group  (Mean ± SD) | P-value |
| --- | --- | --- | --- |
| *IL-1β* | 1.074 ± 0.514 | 3.440 ± 1.444 | 0.0318 |
| *TNF-α* | 1.030 ± 0.292 | 1.644 ± 0.359 | 0.0381 |
| *IL-6* | 1.001 ± 0.058 | 2.179 ± 0.965 | 0.0805 |
| *p16* | 1.099 ± 0.535 | 6.703 ± 2.723 | 0.0112 |
| *P21* | 1.028 ± 0.300 | 2.719 ± 1.448 | 0.0941 |
| *MCP-1* | 1.096 ± 0.499 | 2.674 ± 1.432 | 0.1147 |
| *Tricellulin* | 1.083 ± 0.540 | 0.533 ± 0.094 | 0.0355 |
| *Occludin* | 1.083 ± 0.540 | 0.405 ± 0.266 | 0.0343 |
| *Claudin1* | 1.039 ± 0.325 | 0.853 ± 0.346 | 0.465 |
| *Claudin4* | 1.082 ± 0.459 | 0.681 ± 0.378 | 0.202 |
